# Serum miR-222 is independently associated with atrial fibrillation in patients with degenerative valvular heart disease

**DOI:** 10.1186/s12872-021-01909-7

**Published:** 2021-02-16

**Authors:** Hualan Zhou, Sen Lin, Xia Li, Dianxuan Guo, Yun Wang, Youdong Hu

**Affiliations:** 1 Department of Gereology, Affiliated Huai’an Hospital of Xuzhou Medical University, ’, Huaian, Jiangsu Province, 223002 China; 2 Department of Clinical Laboratory, Affiliated Huai’an Hospital of Xuzhou Medical University, ’, Huaian, Jiangsu Province, 223002 China

**Keywords:** Degenerative valvular heart disease, MicroRNA, Interleukin-6, Brain natriuretic peptide

## Abstract

**Background:**

Inflammation is involved in the progression of degenerative valvular heart disease (DVHD). microRNA-222 (miR-222) contributes to inflammation-mediated vascular remodeling, but its involvement in DVHD in relation to atrial fibrillation (AF) is unknown. This study aimed to investigate the changes in miR-222, interleukin (IL)-6, high-sensitivity C-reactive protein (hs-CRP), and N-terminal pro-brain natriuretic peptide (NT-proBNP) in patients with DVHD complicated with AF.

**Methods:**

This was a case control study of patients with DVHD who were hospitalized at the Geriatrics Department of the Affiliated Huai’an Hospital of Xuzhou Medical University between 01/2017 and 08/2018. The participants were grouped according to the presence of AF, and serum miR-222, IL-6, hs-CRP, and NT-proBNP levels were compared.

**Results:**

There were fifty-two participants (28 males) in the DVHD with AF group, aged 60–80 years (73.0 ± 5.9 years). Sixty participants (31 males) were included in the DVHD without AF group, aged 60–80 years (71.9 ± 6.92 years). There were no significant differences in age, sex, body mass index, fasting blood glucose, triglycerides, cholesterol, and blood pressure between the two groups. The serum levels of miRNA-222, IL-6, hs-CRP, and NT-proBNP in DVHD patients were significantly higher in those with AF compared with the non-AF group (all *P* < 0.05). Correlation analyses revealed that IL-6, hs-CRP, and NT-proBNP levels were positively correlated with miR-222 levels in all patients (IL-6: r = 0.507, *P* < 0.01; hs-CRP: r = 0.390, *P* < 0.01; NT-proBNP: r = 0.509, *P* < 0.01).

**Conclusions:**

Serum miR-222 was independently associated with AF in patients with DVHD.

## Background

Degenerative valvular heart disease (DVHD), also known as calcific aortic valve disease, represents a common heart disease in the elderly and is characterized by the deposition of a large amount of calcium in a cardiac valve. In the United States, the prevalence rates of DVHD is 0.86% in the adult population and 2.8% in people ≥ 75 years of age [1]. DVHD can lead to heart failure, arrhythmia, and even sudden death in the elderly [2–5]. Its risk factors include high levels of von Willebrand factor, congenital abnormality, age, metabolic syndrome, elevated lipoprotein amounts, and the classical risk factors for atherosclerosis [5].

Atrial fibrillation (AF) is a common supraventricular tachyarrhythmia caused by uncoordinated atrial activation and associated with an irregularly irregular ventricular response [6, 7]. Causes of atrial fibrillation include an underlying structural heart disease, metabolic disorders, endocrine diseases, and certain medications [6, 7]. The prevalence of AF is approximately 1–2% in the general population in developed countries [6, 7]. AF may lead to systemic embolization and heart failure [6, 7]. AF is an independent risk factor in patients with valve calcification [8].

Calcific aortic stenosis results from processes similar to atherosclerosis, including lipid accumulation and damage to endothelial cells (e.g., due to stress or radiation) that allows infiltration of lipids into the fibrosa; lipid infiltration and accumulation may recruit inflammatory cells (such as macrophages) into the aortic valve, and bioactive lipid species may be formed due to oxidation, which further promotes inflammation and mineralization of valve leaflets [5]. Inflammation is considered to be involved in the osteogenic processes leading to mineralization and calcification [9]. Matrix metalloproteinases (MMPs) are overexpressed, resulting in remodeling of valve tissue and the accumulation of disorganized fibrous tissue [9]. Various cytokines secreted by inflammatory cells, such as tumor necrosis factor (TNF)-α and interleukin (IL)-6, are involved in molecular pathways leading to mineralization and calcification [9].

Many studies have shown that microRNAs are involved in valve calcification [10, 11]. Studies also revealed that the expression levels of miR-125b and chemokine receptor type 4 (CCR4) are increased in patients with calcific aortic valve disease, suggesting that miRNA and inflammatory responses are important in the development and progression of valvular heart disease [12, 13]. A previous study reported that miR-222 is involved in inflammation-mediated vascular remodeling [14].

Nevertheless, studies assessing the relationship between microRNA-222 (miR-222) and DVHD are rare. The present study aimed to investigate the differences of miR-222, IL-6, high-sensitivity C-reactive protein (hs-CRP), and N-terminal pro-brain natriuretic peptide (NT-proBNP) levels in patients with DVHD with or without AF.

## Methods

### Participants

This was a case control study of patients with DVHD who were hospitalized at the Geriatrics Department of the Affiliated Huai’an Hospital of Xuzhou Medical University between January 2017 and August 2018. Inclusion criteria were: (1) age ≥ 60 years old; (2) diagnosis of DVHD by Philips IE33 color Doppler ultrasonography. DVHD was diagnosed by ultrasound as follows: with the echo of the aortic root’s posterior wall or the mitral annulus’ inferior ventricular wall as an internal reference, the valve or annulus was judged as calcified if the echo of the posterior wall was greater than or equal to that of the reflex of the posterior wall. Specific determination criteria were: (1) aortic valve calcification (aortic valve thickening ≥ 3 mm), enhanced echo, valve lobe stiffness and limited movement; (2) mitral valve and annulus calcification, mitral valve and annulus showing patchy echo enhancement between the posterior wall of the left ventricle and thickness ≥ 3 mm) [15]. Exclusion criteria were: (1) a history of malignant tumor or connective tissue; (2) a history of infectious diseases within the last two weeks (based on medical records or patient reports); (3) medication history of non-steroidal anti-inflammatory drugs, corticosteroids and immunosuppressive drugs (e.g., methotrexate, cyclophosphamide, azathioprine, hydroxychloroquine and salazosulfapyridine), except aspirin, within two weeks. Patients with AF diagnosed using a routine 12-lead electrocardiogram (ECG) or dynamic electrocardiogram (DCG) were assigned to the DVHD with AF group [16]. Then, they were frequency-matched by age and sex with individuals among DVHD patients without AF treated in the same department.

This study was approved by the Medical Ethics Committee of Huai’an Hospital Affiliated to Xuzhou Medical University. All patients provided signed informed consent.

### Serum tests

All the serum tests were performed within 2 days of admission. Fasting antecubital venous blood samples (4 mL) were collected. After centrifugation, serum was collected and stored at −80 °C until analysis. The detection of serum miRNA-222 was performed by real-time polymerase chain reaction (real-time PCR). Total RNA was extracted using a Trizol kit (Invitrogen Inc., Carlsbad, CA, USA). An RNA reverse transcription kit (GeneCopoeia, Rockville, MD, USA) was used to reverse-transcribe the RNA into cDNA for real-time PCR. The amplification conditions were: (1) 95 °C for 5 min, 10 cycles; and (2) 95 °C for 5 s and 60 °C for 31 s, 40 cycles. The melting curve conditions were: (1) 95 °C for 15 s; (2) 60 °C for 30 s; and (3) 95 °C for 15 s. The Ct values were used to represent the expression level of miRNA-222. U6 was used as an internal reference. The relative expression level of miRNA in each sample was calculated by the 2^−ΔΔCt^ method [17].

The detection of serum IL-6 was performed using a quantitative enzyme-linked immunosorbent assay (ELISA) (Xiamen Huijia Biotechnology Co., Ltd., Xiamen, China), according to the manufacturer’s instructions. A RAYTO RT-6000 detector was used for data acquisition. The serum hs-CRP assay was performed using an immunoturbidimetric method on an AU5800 immunoturbidimetric turbidity analyzer (Beckman Coulter, Brea, CA, USA). The reagent kit was provided by LiangChen Bio (Suzhou) Corporation (Suzhou, China). The intra-assay coefficient of variation (CV) was < 5% and the inter-assay CV was < 10%. The normal reference value was ≤ 3 mg/L. The detection of serum NT-proBNP was performed by the chemiluminescence method (bioMerieux, Marcy l’Étoile, France), strictly following the manufacturer’s instructions.

### Data collection and definition

Age, sex, body mass index (BMI), fasting blood glucose, triglycerides (TG), total cholesterol (TC), systolic blood pressure (SBP), diastolic blood pressure (DBP), hypertension, diabetes, and left ventricular ejection fraction (LVEF) were measured, for blood markers, from the same blood sampling as miR-222, hs-CRP, and NT-proBNP, or, for other assessments, within 6 h of blood sampling.

### Statistical analysis

All analyses were performed using SPSS 17.0 (IBM, Armonk, NY, USA) and GraphPad Prism 7.0 (GraphPad Software Inc., San Diego, CA, USA). Continuous data were expressed as mean ± standard deviation and analyzed by the Student t-test (comparisons of two groups). Categorical variables were presented as number (percentage) and analyzed by the chi-square test or Fisher’s exact test, as appropriate. Correlations were analyzed by Pearson correlation analysis. Multivariate logistic regression analysis was performed to assess associations with DVHD combined with AF. Parameters with *P* < 0.05 in univariable analysis were included in multivariate logistic regression analysis by the enter method. *P* < 0.05 was considered statistically significant.

## Results

### Characteristics of the participants

A total of 112 patients were included. Fifty-two patients (28 males and 24 females) were included in the DVHD with AF group; the age range was 60–80 years (73.0 ± 5.9 years). Sixty patients (31 males and 29 females) were included in the DVHD without AF group; the age range was 60–80 years (71.9 ± 6.92 years). There were no significant differences in age, sex, BMI, fasting blood glucose, TG, TC, SBP, and DBP between the two groups. In addition, there were no marked differences between the two groups in the percentages of patients using antiplatelet aggregation agents or anticoagulants, statins, and angiotensin converting enzyme inhibitors or angiotensin II receptor antagonists (ACEI/ARB) (Table 1).

**Table 1:** Characteristics of the participants with DVHD

	Without AF (n = 60)	With AF (n = 52)	P
Age, years, mean ± SD	71.9 ± 6.9	72.9 ± 5.9	0.829
Age > 75, n (%)	21 (35)	22(42)	0.443
Male, n (%)	31 (40.3)	28 (53.8)	0.703
Type of DVHD, n (%)			
Aortic valve	16 (26.7)	19 (36.5)	0.157
Mitral valve	15 (25)	13 (25)	0.175
Aortic valve and mitral valve	29 (48.3)	20 (38.4)	0.134
Body mass index, kg/m^2^, mean ± SD	25.2 ± 2.2	24.6 ± 2.3	0.806
Fasting blood glucose, mmol/L, mean ± SD	5.9 ± 1.1	5.7 ± 1.0	0.825
TG, mmol/L, mean ± SD	1.7 ± 1.0	1.7 ± 1.0	0.813
TC, mmol/L, mean ± SD	4.4 ± 1.1	4.5 ± 1.1	0.820
SBP, mmHg, mean ± SD	129.5 ± 10.2	126.3 ± 13.6	0.792
DBP, mmHg, mean ± SD	68.3 ± 7.0	66.6 ± 6.2	0.855
Hypertension, n (%)	38 (63.3)	35 (67.3)	0.695
Diabetes, n (%)	32 (53.5)	31 (59.6)	0.569
LVEF, %, mean ± SD	54.85 ± 6.96	54.4 ± 9.0	0.843
Use of antiplatelet aggregation agents or anticoagulants, n (%)	58 (96.6)	51 (98.1)	0.554
Use of statins, n (%)	57 (95.0)	50 (96.1)	0.568
Use of ACEI/ARB, n (%)	38 (63.3)	34 (65.4)	0.489

*SD* standard deviation, *TG* triglycerides, *TC* total cholesterol, *SBP* systolic blood pressure, *DBP* diastolic blood pressure, *LVEF* left ventricular ejection fraction, *ACEI* angiotensin converting enzyme inhibitors, *ARB* angiotensin receptor blocker

### Serum miRNA-222, IL-6, hs-CRP, and NT-proBNP levels

**Fig. 1:**
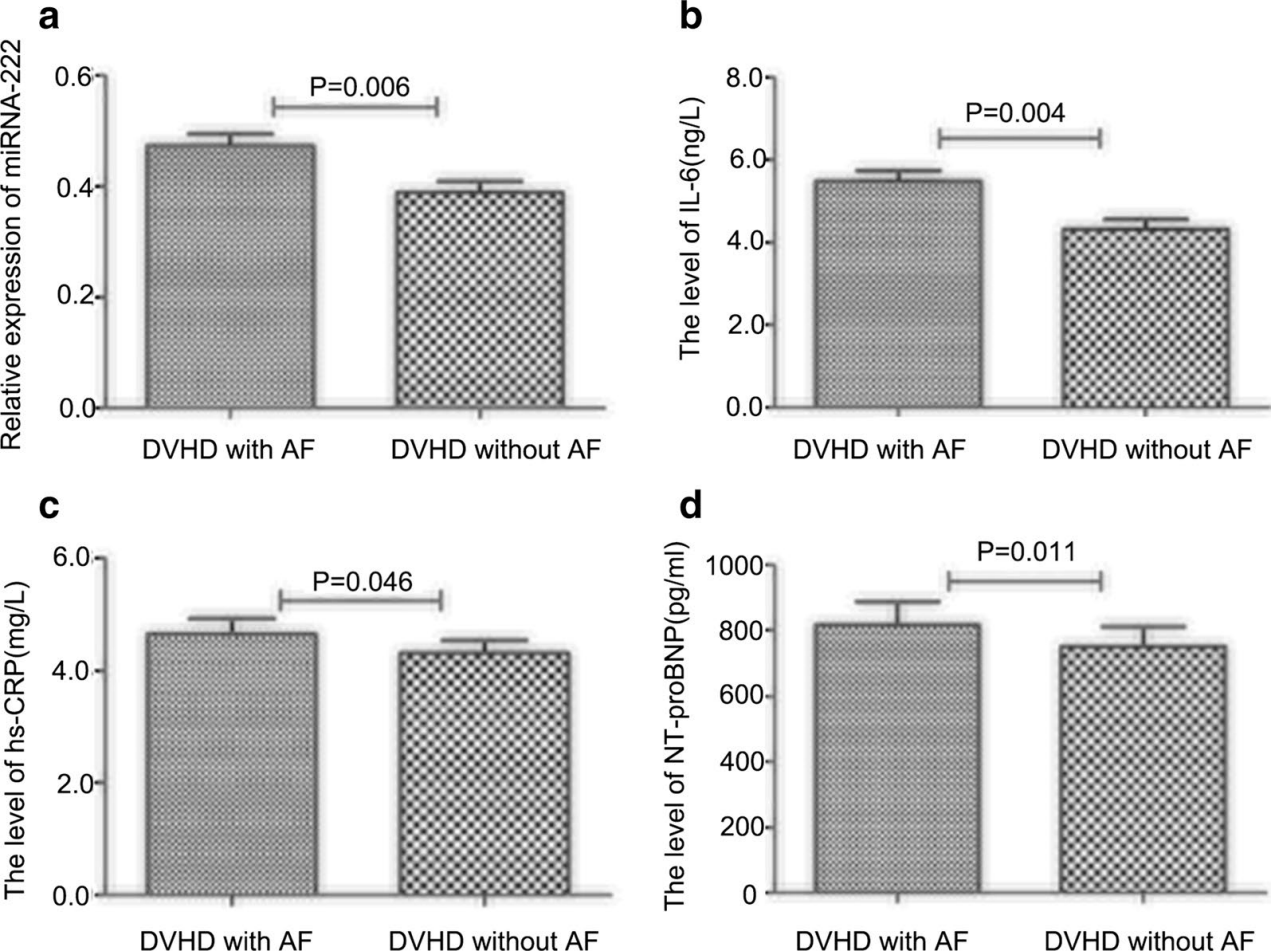
Serum miRNA-222, IL-6, hs-CRP, and NT-proBNP levels. **a** MiRNA-222 levels in the DVHD with AF (0.473 ± 0.154) and DVHD without AF (0.389 ± 0.160) groups (*P* = 0.006). **b** IL-6 levels in the DVHD with AF (5.26 ± 1.68 ng/L) and DVHD without AF (4.31 ± 1.73 ng/L) groups (*P* = 0.004). **c** hs-CRP levels in the DVHD with AF (5.00 ± 1.86 mg/L) and DVHD without AF (4.33 ± 1.65 mg/L) groups (*P* = 0.046). **d** NT-proBNP levels in the DVHD with AF (897.37 ± 452.16 pg/ml) and DVHD without AF (695.22 ± 372.18 pg/ml) groups (*P* = 0.011). IL-6, Interleukin (IL)-6; hs-CRP, high-sensitivity C-reactive protein; NT-proBNP, N-terminal pro b-type natriuretic peptide; DVHD, degenerative valvular heart disease; AF, atrial fibrillation

As shown in Fig. 1, serum levels of miRNA-222, IL-6, hs-CRP, and NT-proBNP in DVHD patients were significantly higher in those with DVHD and AF compared with the non-AF group (all *P* < 0.05).

Multivariate logistic regression analysis was performed with DVHD combined with AF as the dependent variable and serum mirNA-222, IL-6, HS-CRP and NT-proBNP levels as independent variables. The results revealed serum mirNA-222 levels (OR = 64.631, 95%CI: 3.1–1347.6, *P* = 0.007) as an independent predictive factor of DVHD combined AF. Detailed results of the multivariate analysis are shown in Table 2.

**Table 2:** Multivariate logistic regression analysis of serum miRNA-222, IL-6, hs-CRP and NT-proBNP levels in patients with DVHD combined with AF

	OR (95%CI)	*P*
miRNA-222 (2^−△△Ct^)	64.631 (3.1–1347.6)	0.007
IL-6 (ng/L)	1.001 (0.714–1.402)	0.997
hs-CRP (mg/L)	1.075 (0.873–1.324)	0.497
NT-proBNP (pg/ml)	1.000 (0.998–1.001)	0.573

### Correlations

Correlation analyses revealed that IL-6, hs-CRP, and NT-proBNP levels were positively correlated with miR-222 levels in all patients (IL-6: r = 0.507, *P* < 0.01; hs-CRP: r = 0.390, *P* < 0.01; NT-proBNP: r = 0.509, *P* < 0.01).

## Discussion

Inflammation is involved in the progression of DVHD. Meanwhile, miR-222 contributes to inflammation-mediated vascular remodeling, but its involvement in DVHD in relation to AF is unknown. Therefore, this study aimed to investigate the changes in miR-222, IL-6, hs-CRP, and NT-proBNP in patients with DVHD complicated with AF. The results suggest that serum miR-222, IL-6, hs-CRP, and NT-proBNP were associated with AF in patients with DVHD.

MiRNAs are endogenous, highly evolutionarily conserved, single-stranded, non-coding small RNAs [18, 19]. By binding to the 3′UTR regions of target mRNAs, miRNAs regulate gene expression to participate in the occurrence and development of various cellular biological processes such as apoptosis, angiogenesis, inflammation, and ischemic preconditioning [18, 19]. The expression levels of miRNAs vary greatly in different health and disease states, and are often tissue-specific. Detection of the levels of different miRNAs in blood, body fluids, and tissues can be used as a marker for the diagnosis of a given disease and/or a therapeutic target [20, 21]. Studies showed that miR-333 is involved in the development of atherosclerosis in acute coronary syndrome [22]. MiR-126 expression is lower in patients with heart failure and AF [23]. Meanwhile, miR-150 is a predictor of heart failure after myocardial infarction [24]. Blood miR-125, miR-126, miR-21, miR-29b, and miR-30b are downregulated in patients with DVHD [25]. The present study showed that the serum levels of miRNA-222, IL-6, hs-CRP, and NT-proBNP in patients with DVHD with AF were significantly higher compared with those of patients with DVHD without AF.

Next, multivariate logistic regression analysis was performed to assess mirNA-222, IL-6, HS-CRP and NT-proBNP levels for independent associations with DVHD complicated with AF. Of these, only mirNA-222 level was independently associated with DVHD complicated with AF. Other reports have demonstrated the roles of miRNAs in DVHD accompanied by AF. For example, the levels of miRNA-146b-5p and miRNA-155, which are known to be correlated with inflammation, are independently and positively associated with left atrium dimension and atrial fibrillation duration [26]. Another report suggested that miR-10b and miR-138–2, which are significantly increased in the left atrium of individuals with AF-associated rheumatic mitral valve disease (RMVD), are responsible for morphological and physiological phenotype differences between the left and right atria [27].

MiRNAs may participate in the AF regulation process through various mechanisms. For instance, miR-155-5p and miR-24-3p levels are greatly reduced in post-ablation AF patients compared with AF patients not administered ablation [28]. In addition, NO levels were much lower in the AF + group compared with the AF- group. In a swine model of AF, as targets of miR-155-5p and miR-24-3p, eNOS and NO had increased amounts with decreasing miR-155-5p and miR-24-3p amounts, indicating that the above miRNAs are involved in the pathogenesis of AF via eNOS and NO regulation [28]. Meanwhile, miR-21 was detected in atrial myocytes from patients in the sinus rhythm, with significantly greater expression in chronic atrial fibrillation myocytes [29]. In addition, miR-21 showed inverse correlations with α1c subunit of the calcium channel (CACNA1C) gene expression and I(Ca,L) density [29].

The present study also found that IL-6, hs-CRP, and NT-proBNP levels were positively correlated with miR-222 levels in all participants. These findings suggest that miRNA-222, IL-6, hs-CRP, and NT-proBNP might be involved in the exacerbation of DVHD accompanied by AF through the following potential mechanisms. Studies showed that valve degeneration is related to lipid deposition and inflammation [30]. In addition, miR-148a-3p was shown to be involved in the inflammatory response of the valve [31]. The present study found that miR-222, IL-6, and hs-CRP levels were significantly elevated in patients with DVHD, suggesting that inflammatory responses are involved in the development of degenerative valvular disease. Indeed, hs-CRP is a marker of low-grade systemic inflammation, and IL-6 is a proinflammatory cytokine [32, 33]. Investigation of the involvement of miRNAs in the regulation of heart aging through inflammatory responses showed that the miRNA let-7 inhibits angiotensin II-induced cardiomyopathy and fibrosis by inhibiting the expression of IL-6 and multiple collagens in the heart [34]. Patel et al. [35] found that changes in shear-sensitive miRNAs in aortic valve endothelial cells are involved in the development and progression of aortic valve diseases. Rathan et al. [36] showed that miRNA-214 is a shear-dependent miRNA that regulates key mechanosensitive genes related to aortic valve calcification, such as transforming growth factor (TGF)-β1. Animal experiments also showed that the IL-6 signal transduction pathway is involved in the mechanobiological response of aortic valve interstitial cells to promote aortic valve calcification [37]. Studies showed that RNAs affect valve calcification by regulating the levels of osteocalcin [38]. MiR-138 inhibits aortic valve calcification by inhibiting osteogenic differentiation of interstitial valve cells [39]. IL-6 expression is elevated in human calcific aortic valve disease, and phosphate-induced valve mineralization chiefly relies on IL-6 expression [40]. Studies showed that the expression of hypoxia-induced miR-210 is increased in peripheral plasma, monocytes, and skeletal muscle of rats with heart failure [41]. The levels of miRNA-222 in peripheral blood from patients with coronary artery stenosis are increased during dobutamine stress echocardiograms [42]. Therefore, miRNAs participate in DVHD through inflammatory responses, mechanical stress, calcification and osteogenesis, and oxidative stress. Of note, many of those miRNAs are also involved in AF [27–29], and there is a possibility of synergistic effects among those involved in DVHD and AF. Additional transcriptome-wide studies are necessary to identify those relationships.

This study had limitations. First, the sample size was small, and only one miRNA was assessed. Further studies are necessary to examine the roles of miRNAs in DVHD. In addition, the panel of inflammatory markers was limited, and oxidative stress assessment and quantification of valve calcification were not performed. Finally, the clinical history of patients and DVHD severity were not taken into consideration, which might highly bias our findings. Therefore, previous myocardial infarction, stroke, chronic kidney disease and liver disease should be evaluated for their effects on miRNA-222, IL-6 and NT-proBNP.

## Conclusions

Overall, miR222 and inflammatory responses may be involved in the exacerbation of DVHD by AF. Combined detection of miRNA-222, IL-6, and NT-proBNP could assist in the evaluation and clinical management of DVHD.

## Data Availability

The datasets generated and/or analyzed during the current study will not be available to the public due that we assured patients’ privacy would be protected, but are available from the corresponding author on reasonable request. Data sharing is not applicable to this article as no datasets were generated or analyzed during the current study.

## Abbreviations

DVHD: Degenerative valvular heart disease
miR-222: MicroRNA-222
AF: Atrial fibrillation
IL: Interleukin
NT-proBNP: N-terminal pro-brain natriuretic peptide
MMPs: Matrix metalloproteinases
TNF: Tumor necrosis factor
CCR4: Chemokine receptor type 4
hs-CRP: High-sensitivity C-reactive protein
ELISA: Enzyme-linked immunosorbent assay
CV: Coefficient of variation
BMI: Body mass index
TG: Triglycerides
TC: Total cholesterol
SBP: Systolic blood pressure
DBP: Diastolic blood pressure
LVEF: Left ventricular ejection fraction

## References

[1] Nkomo VT, Gardin JM, Skelton TN, Gottdiener JS, Scott CG, Enriquez-Sarano M (2006). Burden of valvular heart diseases: a population-based study. Lancet.

[2] Nishimura RA, Otto CM, Bonow RO, Carabello BA, Erwin JP, Guyton RA (2014). 2014 AHA/ACC guideline for the management of patients with valvular heart disease: a report of the American College of Cardiology/American Heart Association Task Force on Practice Guidelines. Circulation.

[3] Baumgartner H, Falk V, Bax JJ, De Bonis M, Hamm C, Holm PJ (2017). 2017 ESC/EACTS guidelines for the management of valvular heart disease. Eur Heart J.

[4] Grimard BH, Safford RE, Burns EL (2016). Aortic stenosis: diagnosis and treatment. Am Fam Physician.

[5] Lindman BR, Clavel MA, Mathieu P, Iung B, Lancellotti P, Otto CM (2016). Calcific aortic stenosis. Nat Rev Dis Primers.

[6] Kirchhof P, Benussi S, Kotecha D, Ahlsson A, Atar D, Casadei B (2016). ESC Guidelines for the management of atrial fibrillation developed in collaboration with EACTS. Europace.

[7] January CT, Wann LS, Alpert JS, Calkins H, Cigarroa JE, Cleveland JC (2014). 2014 AHA/ACC/HRS guideline for the management of patients with atrial fibrillation: a report of the American College of Cardiology/American Heart Association Task Force on practice guidelines and the Heart Rhythm Society. Circulation.

[8] Burup Kristensen C, Jensen JS, Sogaard P, Carstensen HG, Mogelvang R (2012). Atrial fibrillation in aortic stenosis–echocardiographic assessment and prognostic importance. Cardiovasc Ultrasound.

[9] Hulin A, Hego A, Lancellotti P, Oury C (2018). Advances in pathophysiology of calcific aortic valve disease propose novel molecular therapeutic targets. Front Cardiovasc Med.

[10] Xu HX, Wang Y, Zheng DD, Wang T, Pan M, Shi JH (2017). Differential expression of MicroRNAs in calcific aortic stenosis. Clin Lab.

[11] Xiao X, Zhou T, Guo S, Guo C, Zhang Q, Dong N (2017). LncRNA MALAT1 sponges miR-204 to promote osteoblast differentiation of human aortic valve interstitial cells through up-regulating Smad4. Int J Cardiol.

[12] Ohukainen P, Syvaranta S, Napankangas J, Rajamaki K, Taskinen P, Peltonen T (2015). MicroRNA-125b and chemokine CCL4 expression are associated with calcific aortic valve disease. Ann Med.

[13] Rysa J (2016). Gene expression profiling of human calcific aortic valve disease. Genom Data.

[14] Dentelli P, Rosso A, Orso F, Olgasi C, Taverna D, Brizzi MF (2010). microRNA-222 controls neovascularization by regulating signal transducer and activator of transcription 5A expression. Arterioscler Thromb Vasc Biol.

[15] Qian Y. Ultrasound diagnostics. Fourth Military Medical University Press. 2008.

[16] Huang C, Zhang S, Huang D, Hua W (2018). Current knavledge and management recommendations of atrial fibrillation:2018. Chin J Cardiac Pacing Electrophysiol.

[17] Livak KJ, Schmittgen TD (2001). Analysis of relative gene expression data using real-time quantitative PCR and the 2(-Delta Delta C(T)) Method. Methods.

[18] Witek J, Mohiuddin SS. Biochemistry, Pseudogenes. StatPearls. Treasure Island (FL) 2020. 31751022

[19] O'Brien J, Hayder H, Zayed Y, Peng C (2018). Overview of MicroRNA biogenesis, mechanisms of actions, and circulation. Front Endocrinol (Lausanne).

[20] Hanna J, Hossain GS, Kocerha J (2019). The Potential for microRNA Therapeutics and Clinical Research. Front Genet.

[21] Christopher AF, Kaur RP, Kaur G, Kaur A, Gupta V, Bansal P (2016). MicroRNA therapeutics: discovering novel targets and developing specific therapy. Perspect Clin Res.

[22] Ren J, Ma R, Zhang ZB, Li Y, Lei P, Men JL (2018). Effects of microRNA-330 on vulnerable atherosclerotic plaques formation and vascular endothelial cell proliferation through the WNT signaling pathway in acute coronary syndrome. J Cell Biochem.

[23] Wei XJ, Han M, Yang FY, Wei GC, Liang ZG, Yao H (2015). Biological significance of miR-126 expression in atrial fibrillation and heart failure. Braz J Med Biol Res.

[24] Lin X, Zhang S, Huo Z (2019). Serum Circulating miR-150 is a Predictor of Post-Acute Myocardial Infarction Heart Failure. Int Heart J.

[25] Hulanicka M, Garncarz M, Parzeniecka-Jaworska M, Jank M (2014). Plasma miRNAs as potential biomarkers of chronic degenerative valvular disease in Dachshunds. BMC Vet Res.

[26] Rosjo H, Dahl MB, Bye A, Andreassen J, Jorgensen M, Wisloff U (2014). Prognostic value of circulating microRNA-210 levels in patients with moderate to severe aortic stenosis. PLoS ONE.

[27] van den Berg NWE, Kawasaki M, Berger WR, Neefs J, Meulendijks E, Tijsen AJ (2017). MicroRNAs in atrial fibrillation: from expression signatures to functional implications. Cardiovasc Drugs Ther.

[28] da Silva AM, de Araujo JN, de Freitas RC, Silbiger VN (2017). Circulating MicroRNAs as potential biomarkers of atrial fibrillation. Biomed Res Int.

[29] Jiang S, Guo C, Zhang W, Che W, Zhang J, Zhuang S (2019). The integrative regulatory network of circRNA, microRNA, and mRNA in atrial fibrillation. Front Genet.

[30] Alnabelsi TS, Alhamshari Y, Mulki RH, Codolosa JN, Koshkelashvili N, Goykhman I (2016). Relation between epicardial adipose and aortic valve and mitral annular calcium determined by computed tomography in subjects Aged >/=65 years. Am J Cardiol.

[31] Qin B, Cao Y, Yang H, Xiao B, Lu Z (2015). MicroRNA-221/222 regulate ox-LDL-induced endothelial apoptosis via Ets-1/p21 inhibition. Mol Cell Biochem.

[32] Nehring SM, Goyal A, Patel BC. C Reactive Protein (CRP). StatPearls. Treasure Island (FL)2020.

[33] Justiz Vaillant AA, Qurie A. Interleukin. StatPearls. Treasure Island (FL)2020.

[34] Skarn M, Namlos HM, Noordhuis P, Wang MY, Meza-Zepeda LA, Myklebost O (2012). Adipocyte differentiation of human bone marrow-derived stromal cells is modulated by microRNA-155, microRNA-221, and microRNA-222. Stem Cells Dev.

[35] Patel V, Carrion K, Hollands A, Hinton A, Gallegos T, Dyo J (2015). The stretch responsive microRNA miR-148a-3p is a novel repressor of IKBKB, NF-kappaB signaling, and inflammatory gene expression in human aortic valve cells. FASEB J.

[36] Rathan S, Ankeny CJ, Arjunon S, Ferdous Z, Kumar S, Fernandez Esmerats J (2016). Identification of side- and shear-dependent microRNAs regulating porcine aortic valve pathogenesis. Sci Rep.

[37] Bowler MA, Bersi MR, Ryzhova LM, Jerrell RJ, Parekh A, Merryman WD (2018). Cadherin-11 as a regulator of valve myofibroblast mechanobiology. Am J Physiol Heart Circ Physiol.

[38] Takahashi K, Satoh M, Takahashi Y, Osaki T, Nasu T, Tamada M (2016). Dysregulation of ossification-related miRNAs in circulating osteogenic progenitor cells obtained from patients with aortic stenosis. Clin Sci (Lond).

[39] Lu P, Yin B, Liu L (2019). MicroRNA-138 suppresses osteoblastic differentiation of valvular interstitial cells in degenerative calcific aortic valve disease. Int Heart J.

[40] El Husseini D, Boulanger MC, Mahmut A, Bouchareb R, Laflamme MH, Fournier D (2014). P2Y2 receptor represses IL-6 expression by valve interstitial cells through Akt: implication for calcific aortic valve disease. J Mol Cell Cardiol.

[41] Endo K, Naito Y, Ji X, Nakanishi M, Noguchi T, Goto Y (2013). MicroRNA 210 as a biomarker for congestive heart failure. Biol Pharm Bull.

[42] Jansen F, Schafer L, Wang H, Schmitz T, Flender A, Schueler R, et al. Kinetics of circulating MicroRNAs in response to cardiac stress in patients with coronary artery disease. J Am Heart Assoc. 2017;6. 10.1161/JAHA.116.005270 PMC5586407 28751542

